# Long-term mortality and treatment outcomes in pacemaker-associated heart failure: insights from a nationwide propensity-matched study

**DOI:** 10.1093/ehjopen/oeag027

**Published:** 2026-02-16

**Authors:** Young Jun Park, Sungjoo Lee, Sungjun Hong, Kyunga Kim, Juwon Kim, Ju Youn Kim, Kyoung-Min Park, Young Keun On, Seung-Jung Park

**Affiliations:** Division of Cardiology, Department of Internal Medicine, Wonju Severance Christian Hospital, Yonsei University Wonju College of Medicine, 20 Ilsan-ro, Wonju 26426, Republic of Korea; Department of Digital Health, Samsung Advanced Institute for Health Sciences & Technology, Sungkyunkwan University, 81 Irwon-ro, Seoul 06351, Republic of Korea; Department of Digital Health, Samsung Advanced Institute for Health Sciences & Technology, Sungkyunkwan University, 81 Irwon-ro, Seoul 06351, Republic of Korea; Medical AI Research Center, Research Institute for Future Medicine, Samsung Medical Center, 81 Irwon-ro, Seoul 06351, Republic of Korea; Department of Digital Health, Samsung Advanced Institute for Health Sciences & Technology, Sungkyunkwan University, 81 Irwon-ro, Seoul 06351, Republic of Korea; Biomedical Statistics Center, Research Institute for Future Medicine, Samsung Medical Center, 81 Irwon-ro, Seoul 06351, Republic of Korea; Division of Cardiology, Department of Internal Medicine, Heart Vascular and Stroke Institute, Samsung Medical Center, Sungkyunkwan University School of Medicine, 81 Irwon-ro, Seoul 06351, Republic of Korea; Division of Cardiology, Department of Internal Medicine, Heart Vascular and Stroke Institute, Samsung Medical Center, Sungkyunkwan University School of Medicine, 81 Irwon-ro, Seoul 06351, Republic of Korea; Division of Cardiology, Department of Internal Medicine, Heart Vascular and Stroke Institute, Samsung Medical Center, Sungkyunkwan University School of Medicine, 81 Irwon-ro, Seoul 06351, Republic of Korea; Division of Cardiology, Department of Internal Medicine, Heart Vascular and Stroke Institute, Samsung Medical Center, Sungkyunkwan University School of Medicine, 81 Irwon-ro, Seoul 06351, Republic of Korea; Division of Cardiology, Department of Internal Medicine, Heart Vascular and Stroke Institute, Samsung Medical Center, Sungkyunkwan University School of Medicine, 81 Irwon-ro, Seoul 06351, Republic of Korea

**Keywords:** Pacemaker- or pacing-associated heart failure, Mortality, CRT upgrade, Angiotensin receptor–neprilysin inhibitor, Nationwide cohort

## Abstract

**Aims:**

Pacing-associated heart failure (PaHF) has emerged as a clinically significant complication in patients with pacemakers, yet its prognostic factors and optimal management remain underexplored. We aimed to assess mortality risk and the clinical impact of cardiac resynchronisation therapy (CRT)-upgrade and heart failure (HF) medications in patients with PaHF using a nationwide real-world cohort.

**Methods and results:**

We analysed 4166 patients who developed PaHF after *de novo* permanent pacemaker implantation using a nationwide real-world cohort from the Korean National Health Insurance Service. To address confounding and immortal-time bias, propensity score matching and time-dependent Cox regression models were applied. During a median follow-up of 1.9 years, 330 patients underwent CRT-upgrade in addition to standard HF medical therapy, while 3836 received guideline-directed HF medications alone. Increasing age (HR = 1.05 per year, 95% CI 1.04–1.06, *P* < 0.001), male sex (HR = 1.41, 95% CI 1.16–1.71, *P* < 0.001), diabetes (HR = 1.28, 95% CI 1.02–1.60, *P* = 0.035), and chronic kidney disease or end-stage renal disease (HR = 1.69, 95% CI 1.32–2.17, *P* < 0.001) were independently associated with increased all-cause mortality. In contrast, CRT-upgrade (HR = 0.36, 95% CI 0.26–0.50, *P* < 0.001), angiotensin receptor–neprilysin inhibitor (ARNI) use (HR = 0.37, 95% CI 0.19–0.68, *P* = 0.004), and beta-blockers (HR = 0.80, 95% CI 0.64–0.99, *P* = 0.042) were strongly associated with improved survival.

**Conclusion:**

In this nationwide real-world cohort, CRT-upgrade was associated with a significant reduction in all-cause mortality compared with medical therapy alone in patients with PaHF. These findings support the prognostic importance of device-based therapy in combination with contemporary HF medical treatment in real-world clinical practice.

## Graphical Abstract

**Graphical Abstract:**
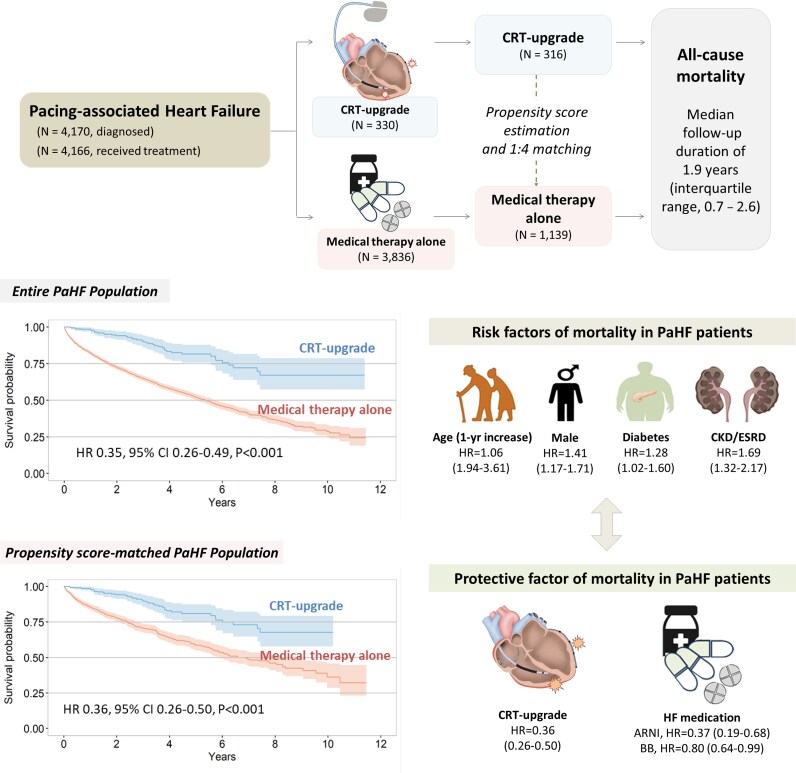
Among 4166 patients diagnosed with PaHF after *de novo* pacemaker implantation between 2008 and 2019, 330 received a CRT-upgrade, while 3836 were treated with standard HF medications alone. After 1:4 propensity score matching, 316 patients in the CRT-upgrade group were compared to 1139 matched controls without CRT-upgrade. The primary outcome was all-cause mortality, with a median follow-up duration of 1.9 years (interquartile range, 0.7–2.6 years). CRT-upgrade was associated with significantly improved survival in the matched cohorts (HR 0.36, 95% CI 0.26–0.50, *P* < 0.001). Multivariable analysis identified increasing age, male sex, diabetes, and CKD or ESRD as independent risk factors for mortality, whereas CRT-upgrade and use of ARNI or beta-blockers were protective. Abbreviations: ARNI, angiotensin receptor neprilysin inhibitor; CKD, chronic kidney disease; CRT, cardiac resynchronisation therapy; ESRD, end-stage renal disease; HF, heart failure; PaHF, pacemaker-associated heart failure; PPM, permanent pacemaker.

## Introduction

The clinical significance of pacing- or pacemaker-associated heart failure (PaHF) is gaining attention due to its association with adverse outcomes, including chronic ventricular pacing-induced left ventricular (LV) dysfunction, increased mortality, and HF hospitalisation, particularly in pacing-dependent patients with new-onset LV systolic dysfunction.^1–3^ Chronic right ventricular (RV) pacing, while central to permanent pacemaker (PPM) therapy, may induce electromechanical dyssynchrony and contribute to the development of PaHF.^1,4–6^

Although cardiac resynchronisation therapy (CRT) has shown potential benefit in selected patients with PaHF,^7,8^ the optimal timing of device-based intervention and appropriate patient selection remain uncertain. Moreover, most previous studies on PaHF were single-centre analyses with relatively small sample sizes, limiting the generalisability and statistical power.^1–5^ In addition, evidence guiding the medical management of PaHF is limited. It is unknown whether standard HF pharmacotherapy alone can improve outcomes in PaHF or whether device-based therapy is essential for modifying its natural history. In particular, the role of newer agents such as angiotensin receptor–neprilysin inhibitors (ARNIs) in PaHF has not been adequately explored, and few studies have rigorously compared CRT-upgrade vs. medical therapy alone using robust statistical methods.

To address these gaps, we conducted a nationwide cohort study involving more than 4000 patients with newly diagnosed PaHF in South Korea. By leveraging a propensity score (PS)–matched cohort and employing time-dependent analyses to account for immortal-time bias, we aimed to evaluate the prognostic impact of CRT-upgrade in real-world patients with PaHF. In addition, we explored the survival effects of contemporary HF pharmacotherapies, including ARNIs and beta-blockers, to assess whether optimized medical treatment could complement or potentially substitute device-based intervention.

## Methods

### Data sources

This nationwide retrospective cohort study utilized data from the National Health Information Database (NHID), managed by the Korean National Health Insurance Service (NHIS). The NHIS is a mandatory, single-payer social health insurance system overseen by the Korean government, covering nearly the entire South Korean population (over 52 million individuals).^9^ The NHID contains comprehensive healthcare information, including demographics, diagnoses [based on the International Classification of Diseases, 10th Revision (ICD-10)], hospital visits and admissions, prescription records (using Anatomical Therapeutic Chemical codes), procedures, medical device usage, and mortality data. Established nationwide in 2000, the database has been accessible for academic and policy research purposes via the NHIS Data Sharing Service (http://nhiss.nhis.or.kr) since 2009.^10,11^ The validity of the diagnostic codes in the NHID has been previously demonstrated, showing high accuracy for major cardiovascular or intractable diseases, such as hypertrophic cardiomyopathy (93%) or myocardial infarction (92%).^9,12,13^ This study was approved by the Institutional Review Board (IRB) of Samsung Medical Center. The requirement of informed consent was waived as all data were deidentified and publicly available under strict confidentiality protocols.

### Data acquisition

Demographic data such as age and sex were obtained. Comorbidities were identified based on ICD-10 codes from claims data spanning one year prior to the index date through the date of PaHF diagnosis (see Supplementary material online, *Table S1*). The Charlson comorbidity index (CCI), representing the overall comorbidity load at baseline, was calculated based on CCI-related variables with their ICD-10 codes (see Supplementary material online, *Table S2*). We identified medication history for renin-angiotensin system (RAS) inhibitors, including angiotensin-converting-enzyme inhibitors (ACEIs), angiotensin II receptor blockers (ARBs), and ARNI. Additional drug classes, such as beta-blockers, mineralocorticoid receptor antagonists (MRAs), loop diuretics, thiazides, antiplatelet agents, and anticoagulants, were also retrieved. Pacemaker type and CRT-upgrade were defined using ICD-10 codes for cardiac implantable electronic device and procedure (see Supplementary material online, *Table S3*).

### Study population and outcomes

The study population included adults (≥18 years) who underwent *de novo* PPM implantation and subsequently developed PaHF between 1 January 2008 and 31 December 2019, using the procedure and device codes for claims reimbursement (see Supplementary material online, *Table S3*). Because the present study was based on a nationwide administrative claims database, PaHF was operationally defined using clinically actionable diagnostic and treatment triggers to minimize misclassification. Specifically, PaHF was strictly defined as (1) hospitalisation with a newly assigned HF code (I50.9) following PPM implantation together with claims for ≥2 different guideline-directed HF medication classes among ACEIs/ARBs, beta-blockers, or MRAs, or (2) initiation of ARNI therapy or receipt of CRT-upgrade after PPM implantation, regardless of HF diagnostic code assignment or concomitant HF medication use. The inclusion of ARNI initiation and CRT-upgrade as qualifying criteria for PaHF was based on their strict reimbursement requirements within the Korean healthcare system, under which ARNI prescription is limited to patients with LVEF <40% and CRT-upgrade is reimbursed only for those with LVEF <35%, thereby requiring objective documentation of advanced systolic dysfunction and clinically significant HF following PPM implantation.

Patients with a history of HF prior to PPM implantation were excluded. Prior HF history was defined as hospitalisation with HF code (I50.9) or prescription history of ARNI prior to PPM implantation. Furthermore, to ensure that the HF was truly pacing-associated, patients were excluded from the PaHF cohort if they had been diagnosed, prior to fulfilling the PaHF criteria, with alternative conditions known to independently cause HF. These included hospitalized myocardial infarction (ICD-10 code I21) or myocardial infarction treated with percutaneous coronary intervention, identified by diagnosis codes I21, I22, or I23 in conjunction with a coronary angiography procedure code (HA670); myocarditis, defined by hospitalisation with ICD-10 codes I01.2, I09.0, I40.0–I40.9, I41.0–I41.8, or I51.4; alcoholic cardiomyopathy (ICD-9 code 425.5 or ICD-10 code I42.6); cardiac sarcoidosis (ICD-10 codes V111 and D860–D863, D868, D869); and cardiac amyloidosis, including transthyretin (TTR) or amyloid light chain (AL) subtypes (ICD-10 code E85). These conditions were regarded as alternative etiologies of heart failure unrelated to chronic right ventricular pacing and were therefore excluded to maintain the specificity of the PaHF cohort.

The primary outcome was all-cause mortality. Follow-up duration for all-cause mortality was calculated from the date of PaHF diagnosis to either death or the last available follow-up, whichever occurred first.

#### Statistical analyses

Baseline characteristics were described as means with standard deviations for continuous variables, and as counts with percentages for categorical variables. Continuous variables were compared using a Student’s *t*-test or Mann–Whitney U test, whereas categorical variables were compared using the χ2 test or Fisher's exact test, as appropriate.

**Figure 1:**
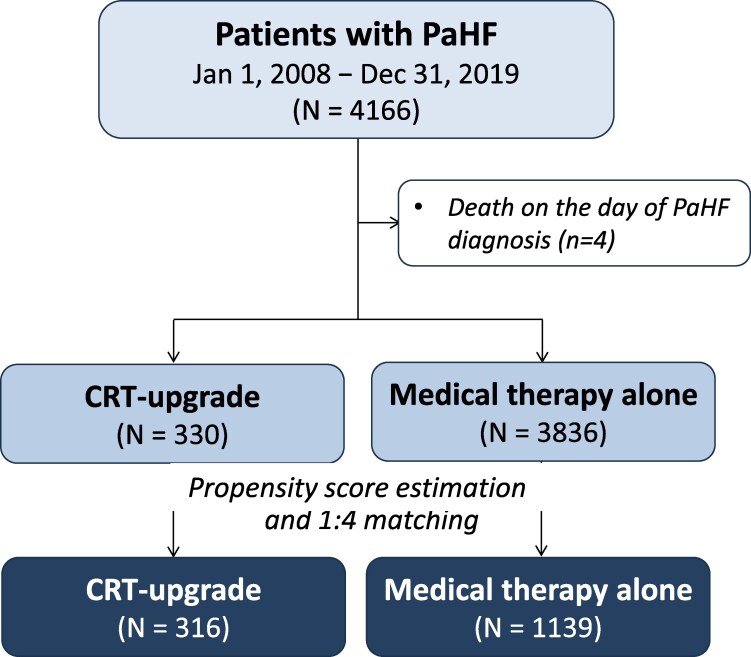
Study flowchart of patient selection and propensity score-matched cohorts. A total of 4166 patients diagnosed with PaHF between 1 January 2008 and 31 December 2019 were identified from the nationwide registry. After excluding 4 patients who died on the day of PaHF diagnosis, 330 patients underwent CRT-upgrade, and 3836 did not. Propensity score matching was performed, yielding 316 CRT-upgrade patients matched to 1139 patients without CRT-upgrade. Abbreviations: cardiac resynchronisation therapy, CRT; PaHF, pacemaker-associated heart failure; PPM, permanent pacemaker.

Incidence rates of all-cause mortality were calculated as the number of deaths per 100 patient-years (PYs), and exact 95% confidence intervals (CIs) were derived using the Poisson distribution. To assess the impact of CRT-upgrade on mortality, patients with PaHF were stratified into CRT-upgrade and medical therapy alone groups. To emulate a randomized controlled trial, we constructed a PS-matched cohort of PaHF patients with a 1:4 ratio (*Figure 1*). The PS, defined as the probability of receiving CRT-upgrade, was estimated using a multivariable binary logistic regression model based on relevant clinical covariates. Matching was performed using greedy nearest-neighbour matching without replacement, with a calliper width equal to 0.25 times the pooled standard deviation of the logit of the PS. Standardized mean differences (SMDs) were used for balance diagnostics, and absolute values of SMD > 0.1 were considered indicative of meaningful imbalance.

The date of PaHF diagnosis was considered as time-zero for all survival analyses. Kaplan–Meier survival curves were generated to estimate cumulative survival in both the entire PaHF cohort and the PS-matched cohort. Comparisons between groups were performed using the log-rank test for the overall cohort and the stratified log-rank test for the PS-matched cohort, respectively. However, since the CRT-upgrade was initiated during the follow-up period rather than at baseline, the primary analysis could be susceptible to immortal-time bias (see Supplementary material online, *Figure S1*). To address this concern, a sensitivity analysis was performed in which CRT-upgrade was modelled as a time-dependent covariate. Extended Kaplan–Meier methods and time-dependent multivariable Cox regression models were employed to estimate adjusted hazard ratios (HRs) with 95% CIs, accounting for the time interval between PaHF diagnosis and CRT-upgrade.

Multivariable Cox proportional hazards (PH) models were used to evaluate the associations between the CRT-upgrade status and all-cause mortality in both cohorts. In the PS-matched cohort, robust standard errors were calculated by treating matched pairs as clusters in the matched cohort. The PH assumption was assessed based on scaled Schoenfeld residuals.

For multivariable analysis, variables with *P* < 0.05 in univariable analyses and those known to be clinically relevant (e.g. age, sex) were selected. Multicollinearity was assessed by the variance inflation factor (VIF), with VIF > 4 indicating significant collinearity. All statistical analyses were performed using SAS version 9.3 (SAS Institute, Cary, NC, USA) and R version 4.1.0 (The R Foundation, www.R-project.org). Two-sided *P* values < 0.05 were considered statistically significant.

## Results

### Study population

During the study period, a total of 4170 patients met the criteria for PaHF. After excluding 4 who died on the day of PaHF diagnosis, 4166 patients were included in the final analysis. Among them, 330 patients received CRT-upgrade in addition to standard HF therapy, while 3836 received medical therapy alone without CRT-upgrade (*Figure 1*).

Baseline characteristics of the CRT-upgrade and medical therapy alone groups are summarized in *Table 1*. Prior to PS-matching, patients who underwent CRT-upgrade were generally younger, had a lower burden of comorbidities (e.g. atrial fibrillation, coronary artery disease, and CKD/ESRD), and more frequently had pacemaker indications related to atrioventricular block. Medication use patterns were also different between groups. After PS-matching (316 CRT-upgrade vs. 1139 medical therapy alone patients), the baseline characteristics between groups were well balanced, with most absolute SMD < 0.2. Residual imbalances remained only for age (mean difference: 0.3 years, aSMD = 0.196), use of ARNI (aSMD = 0.212), and loop diuretic use (aSMD = 0.219), but these differences were considered modest.

**Table 1:** Baseline characteristics of patients with PaHF

Variables	Entire PaHF cohort			PS-matched PaHF cohort		
	CRT-upgrade^a^ (*n* = 330)	Medical therapy alone^a^ (*n* = 3836)	aSMD	CRT-upgrade^a^ (*n* = 316)	Medical therapy alone^a^ (*n* = 1139)	aSMD
***Demographics and medical history***						
Age, years	67.5 ± 13.7	75.6 ± 10.5	0.670	67.7 ± 13.4	70.3 ± 12.4	0.196
Age ≥ 65 years	215 (65.2)	3313 (86.4)	0.511	207 (65.5)	817 (71.7)	0.131
Male	155 (47.0)	1658 (43.2)	0.075	149 (47.2)	524 (46.0)	0.022
Diabetes	89 (27.0)	1270 (33.1)	0.134	84 (26.6)	342 (30.0)	0.078
Hypertension	247 (74.8)	2944 (76.7)	0.044	236 (74.7)	895 (78.6)	0.089
Coronary artery disease	183 (55.5)	2725 (71.0)	0.328	174 (55.1)	651 (57.2)	0.042
Peripheral artery disease	113 (34.2)	1573 (41.0)	0.140	111 (35.1)	418 (36.7)	0.033
CKD/ESRD	39 (11.8)	720 (18.8)	0.194	39 (12.3)	168 (14.7)	0.073
Valvular heart disease	60 (18.2)	742 (19.3)	0.030	54 (17.1)	176 (15.5)	0.043
Atrial fibrillation	116 (35.2)	1881 (49.0)	0.284	116 (36.7)	439 (38.5)	0.038
COPD	117 (35.5)	1793 (46.7)	0.231	111 (35.1)	419 (36.8)	0.035
***Pacemaker-related variables***						
AV block	231 (70.0)	2284 (59.5)	0.255	226 (71.5)	790 (69.4)	0.048
Sinus node disease	90 (27.3)	1524 (39.7)		90 (28.5)	349 (30.6)	
Dual chamber	289 (87.1)	2843 (73.9)	0.338	276 (87.3)	987 (86.7)	0.021
***Medications***						
RAS inhibitors^b^	220 (66.7)	2425 (63.2)	0.072	209 (66.1)	786 (69.0)	0.061
ARNI	21 (6.4)	296 (7.7)	0.053	19 (6.0)	126 (11.1)	0.212
Beta blockers	151 (45.8)	1964 (51.2)	0.109	141 (44.6)	523 (45.9)	0.026
MRAs	106 (32.1)	1121 (29.2)	0.063	97 (30.7)	359 (31.5)	0.018
Loop diuretics	146 (44.2)	2151 (56.1)	0.238	135 (42.7)	610 (53.6)	0.219
Thiazide	17 (5.2)	211 (5.5)	0.016	17 (5.4)	52 (4.6)	0.036

Values were expressed as means ± standard deviations or *n* (%).

^a^‘CRT-upgrade’ and ‘Medical therapy alone’ represent patients who were treated with CRT-upgrade plus HF medications and those with HF medications alone without CRT-upgrade, respectively.

^b^RAS inhibitors included ACEIs, ARBs, and ARNI.

Abbreviations: PaHF, pacemaker-associated heart failure; PS, propensity score; CRT, cardiac resynchronization therapy; aSMD, absolute standardized mean difference; CKD, chronic kidney disease; ESRD, end-stage renal disease; COPD, chronic obstructive pulmonary disease; AV block, atrioventricular block; ACEIs, angiotensin-converting enzyme inhibitors; ARBs, angiotensin II receptor antagonists; ARNI, angiotensin receptor neprilysin inhibitor; MRAs, mineralocorticoid receptor antagonists.

#### Prognosis according to CRT-upgrade

**Figure 2:**
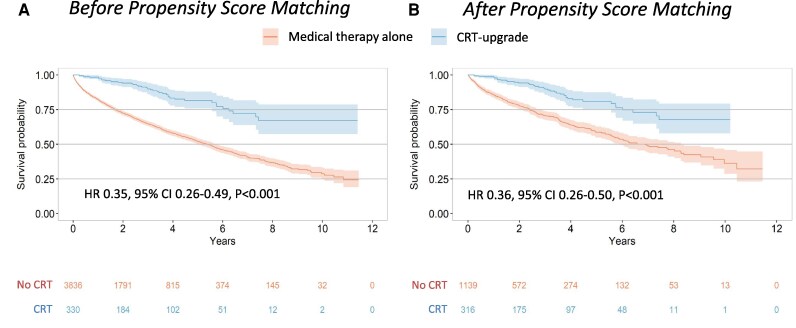
Kaplan–meier survival curves for all-cause death. The long-term prognosis was significantly improved for patients who received CRT-upgrade in combination with medical treatment compared to those who received medical treatment alone without CRT-upgrade. Abbreviations: CRT, cardiac resynchronisation therapy; PaHF, pacemaker-associated heart failure.

During the median follow-up period of 1.9 (interquartile range, 0.7–2.6) years, patients who received CRT-upgrade exhibited significantly lower all-cause mortality compared to those who did not, in both the entire and PS-matched cohorts. In the entire cohort, the incidence rate of all-cause death was 4.1 per 100 PYs (95% CI, 3.0–5.5) in the CRT-upgrade group vs. 14.6 per 100 PYs (95% CI, 13.8–15.3) in the medical therapy alone group (*P* < 0.001). In the PS-matched cohorts, the corresponding rates were 4.1 (95% CI, 3.0–5.5) vs. 11.4 (95% CI, 10.3–12.6) per 100 PYs (*P* < 0.001). Kaplan–Meier survival analysis showed a significantly improved survival probability in the CRT-upgrade group compared to the medical therapy alone group in both the full cohort (log-rank *P* < 0.001) and the PS-matched cohort (stratified log-rank *P* < 0.001) (*Figure 2A* and *B*).

In multivariable Cox regression analyses (*Table 2*), CRT-upgrade remained the most robust protective factor against mortality. In model 1, where RAS inhibitors were treated as a single variable, CRT-upgrade was associated with an adjusted HR of 0.36 (95% CI, 0.26–0.50; *P* < 0.001). Other independent risk factors for mortality included age (per 1-year increase), male sex, DM, and CKD/ESRD, while RAS inhibitor use and beta-blockers were associated with reduced risk. However, when RAS inhibitors were separated into ACEIs/ARBs and ARNI in model 2, only ARNI use emerged as a significant independent protective factor (HR 0.37, 95% CI, 0.19–0.68; *P* = 0.004), whereas ACEIs/ARBs were not independently associated with improved survival (HR 0.86, 95% CI, 0.64–1.08; *P* = 0.199).

**Table 2:** Independent risk factors of all-cause mortality in the propensity score-matched PaHF cohort

Variable	*Univariable analyses*		*Multivariable analysis model 1*		*Multivariable analysis model 2*	
	HR (95% CI)	*P* value	HR (95% CI)	*P* value	HR (95% CI)	*P* value
CRT-upgrade	0.36 (0.26 to 0.49)	<0.001	0.37 (0.26 to 0.51)	<0.001	0.36 (0.26 to 0.50)	<0.001
Age (per 1-year increase)	1.05 (1.04 to 1.06)	<0.001	1.05 (1.04 to 1.06)	<0.001	1.05 (1.04 to 1.06)	<0.001
Male	1.19 (0.99 to 1.44)	0.063	1.38 (1.13 to 1.68)	0.001	1.41 (1.16 to 1.71)	0.001
Diabetes mellitus	1.45 (1.18 to 1.77)	<0.001	1.28 (1.02 to 1.60)	0.034	1.28 (1.02 to 1.60)	0.035
Hypertension	0.60 (0.47 to 0.77)	<0.001	0.48 (0.36 to 0.66)	<0.001	0.44 (0.32 to 0.60)	<0.001
Coronary artery disease	1.21 (0.99 to 1.48)	0.063				
Peripheral artery disease	1.20 (0.97 to 1.48)	0.098				
CKD/ESRD	2.22 (1.78 to 2.77)	<0.001	1.73 (1.35 to 2.21)	<0.001	1.69 (1.32 to 2.17)	<0.001
Valvular heart disease	0.76 (0.58 to 1.00)	0.048	1.06 (0.79 to 1.41)	0.703	1.04 (0.78 to 1.39)	0.773
Atrial fibrillation	0.72 (0.57 to 0.91)	0.006	0.88 (0.67 to 1.14)	0.318	0.90 (0.69 to 1.17)	0.441
COPD	1.38 (1.13 to 1.69)	0.002	1.14 (0.93 to 1.38)	0.200	1.13 (0.93 to 1.37)	0.219
RAS inhibitors^a^	0.59 (0.48 to 0.73)	<0.001	0.73 (0.58 to 0.91)	0.005		
ACEIs or ARBs	0.71 (0.57 to 0.87)	0.001			0.86 (0.64 to 1.08)	0.199
ARNI^†^	0.30 (0.16 to 0.55)	<0.001			0.37 (0.19 to 0.68)	0.004
Beta blockers	0.66 (0.54 to 0.82)	<0.001	0.79 (0.63 to 0.98)	0.034	0.80 (0.64 to 0.99)	0.042
MRAs	1.14 (0.91 to 1.41)	0.248				

^a^RAS inhibitors included ACEIs, ARBs, and ARNI. The variable of RAS inhibitors was included in the Multivariable analysis model 1 as a single composite variable, while ACEIs/ARBs and ARNI were separately included in the Multivariable analysis model 2.

Abbreviations: PaHF, pacemaker-associated heart failure; PS, propensity score; HR, hazard ratio; CI, confidence interval; CRT, cardiac resynchronization therapy; CKD, chronic kidney disease; ESRD, end-stage renal disease; COPD, chronic obstructive pulmonary disease; ACEIs, angiotensin-converting enzyme inhibitors; ARBs, angiotensin II receptor antagonists; ARNI, angiotensin receptor neprilysin inhibitor; MRAs, mineralocorticoid receptor antagonists; AVB, atrioventricular block; SND, sinus node dysfunction; PPM, permanent pacemaker.

#### Sensitivity analyses

Although the median interval from PaHF diagnosis to CRT-upgrade was 0.4 days (IQR 0.4–85.2 days), suggesting a minimal risk of immortal-time bias, we conducted additional analyses to formally account for this possibility. Even after adjusting for immortal-time bias, CRT-upgrade remained significantly associated with reduced all-cause mortality in the PS-matched cohort (HR 0.49, 95% CI 0.34–0.68, *P* < 0.001; Supplementary material online, *Table S4*). This finding was consistent across two models: one including RAS inhibitors as a composite variable and another separating ACEIs/ARBs and ARNI. The protective association of ARNI use was preserved (HR 0.39, 95% CI 0.20–0.76, *P* = 0.005), while ACEIs/ARBs showed no significant effect. Extended Kaplan–Meier survival curves reflecting the time-dependent nature of CRT-upgrade (see Supplementary material online, *Figure S2A* and *S2B*) further corroborate the survival benefit, with significantly improved outcomes in both the full and matched cohorts (log-rank *P* < 0.001 for both).

## Discussion

### Main findings and merits of this study

Our main findings were: (1) CRT-upgrade was associated with a substantial reduction in all-cause mortality compared with standard HF therapy alone in this large real-world cohort of patients with PaHF. This survival benefit was consistently observed in both the entire and PS-matched cohorts and remained robust in multivariable analyses, even after accounting for immortal-time bias through time-dependent modeling; (2) among all evaluated clinical and treatment-related factors, CRT-upgrade emerged as the strongest independent protective factor against mortality, consistently demonstrating the lowest adjusted hazard ratios (adjusted HRs ranging from 0.36 to 0.49) across all multivariable models; and (3) the use of RAS inhibitors, and beta-blockers were also associated with improved survival. However, when RAS inhibitors were evaluated separately, only ARNI use—unlike ACEIs or ARBs—was independently associated with better outcomes.

Our study has several merits compared to previous studies. First, this study was based on one of the largest PaHF cohorts to date. This was established using nationwide, unselected, real-world data that encompasses almost the entire South Korean population, potentially enhancing the generalisability of our findings. Second, in contrast to prior studies (^1,3^ we accounted for immortal-time bias by incorporating CRT-upgrade as a time-dependent covariate, strengthening internal validity. Third, to our knowledge, this is the first nationwide study to concurrently evaluate both the effect of CRT-upgrade and the impact of contemporary HF medications, particularly ARNI, on mortality in patients with PaHF, using a PS-matched design.

### Prognostic factors in PaHF patients

Consistent with previous reports, CKD/ESRD, male sex, DM, and advanced age were independently associated with higher mortality in patients with PaHF (*Table 2*). In CKD, several factors, including ventricular hypertrophy, myocardial fibrosis, and uremia, decelerate myocardial conduction velocity,^14,15^ thereby aggravating pacing-induced dyssynchrony. Furthermore, CKD and ESRD have been reported as independent predictors of PaHF and mortality in patients with PPM.^3,16,17^ Compared to women, men are more likely to have a larger heart size and greater myocardial mass, which can lead to prolonged conduction time and more severe pacing-induced dyssynchrony, ultimately resulting in higher mortality.^18,19^ Worse prognosis in male PaHF patients with larger hearts may reflect the counterpart phenomenon of better CRT responses observed in female patients with smaller hearts.^20^ The specific role of DM in the development or progression of PaHF remains underexplored. However, DM is frequently associated with advanced age, CKD/ESRD, and coronary artery disease, which could potentially amplify mortality risk.^21,22^

### Management implications of CRT-upgrade

After a PaHF diagnosis, the optimal timing of CRT-upgrade remains uncertain, and whether a period (e.g. 3–6 months) of guideline-directed medical therapy (GDMT) should precede CRT-upgrade continues to be debated. Previous studies in patients with HF have suggested that the response to medical therapy alone may be limited in those with electrical dyssynchrony, including left bundle branch block or pacing-induced dyssynchrony, challenging current guidelines that mandate at least three months of GDMT before CRT implantation.^7,23^ In our study, MRAs and ACEIs/ARBs had no significant effect on mortality, whereas patients who received CRT-upgrade demonstrated substantially better survival than those treated with medical therapy alone. Kaplan–Meier analyses showed that survival curves between the two groups began to diverge relatively early after PaHF diagnosis (*Figure 2*).

Notably, in addition to CRT-upgrade, ARNI and beta-blockers were significantly associated with improved survival (*Table 2*), consistent with contemporary guidelines recommending prioritized use of ARNI over ACEIs/ARBs in patients with HFrEF.^24,25^ Therefore, once PaHF has developed, continuation of medical therapy without device upgrade may be insufficient to modify prognosis. CRT-upgrade in combination with contemporary HF medical therapy, preferably including ARNI and beta-blockers, may be warranted in appropriately selected patients. However, the present study was not designed to compare early vs. delayed CRT-upgrade strategies; rather, our findings should be interpreted as evidence supporting the prognostic benefit of CRT-upgrade compared with medical therapy alone.

### Limitations

We acknowledge several limitations. First, because this study was based on nationwide administrative claims data, detailed clinical information such as echocardiographic parameters, device interrogation data (RV-pacing burden), HF phenotype (HFrEF vs. HF with preserved EF), rhythm status, and the speciality of the diagnosing physician was not available. Accordingly, we could not definitively confirm that all cases classified as PaHF were driven solely by pacing-induced electromechanical dyssynchrony, and other contributors to post-PPM HF, such as atrial fibrillation with suboptimal rate control or uncontrolled hypertension, could not be fully excluded. Therefore, some degree of etiological heterogeneity and diagnostic uncertainty are unavoidable and should be considered when interpreting our findings. Nevertheless, we sought to enhance specificity by carefully excluding patients with major alternative etiologies of HF that are unlikely to be pacing-related, including incident myocardial infarction, myocarditis, alcoholic cardiomyopathy, and infiltrative cardiomyopathies such as sarcoidosis or amyloidosis. In addition, within the Korean healthcare system, reimbursement for CRT-upgrades requires objective documentation of (1) LVEF ≤35%, (2) New York Heart Association class III or ambulatory IV, and (3) an RV-pacing percentage ≥ 40% confirmed by device interrogation. These stringent criteria indirectly support the presence of clinically significant pacing-induced cardiomyopathy in many patients who underwent CRT-upgrade. Moreover, the observed survival benefit following CRT-upgrade lends further support to this interpretation. Second, we did not differentiate between CRT with defibrillator (CRT-D) and CRT with pacemaker only (CRT-P) among patients who underwent CRT-upgrade. As a result, we could not evaluate whether the observed survival benefit associated with CRT-upgrade was influenced by the presence of defibrillator therapy. Therefore, the potential differential prognostic impact of CRT-D vs. CRT-P could not be assessed in this study and warrants further investigation in future studies. Third, the clinical decision-making process regarding continuation or escalation of GDMT vs. CRT-upgrade could not be assessed using claims data, and patients undergoing CRT-upgrade may represent a selected subset. To mitigate measured confounding, we performed PS-matching and multivariable adjustment; however, residual selection bias inherent to observational data cannot be completely excluded. In addition, because PaHF was defined using treatment- and hospitalisation-based criteria, the timing of formal PaHF identification may not precisely reflect the true clinical onset of PaHF in all patients. Consequently, the observed interval between PaHF diagnosis and CRT-upgrade may have been underestimated in some cases. Fourth, although our analysis showed a stronger protective effect of ARNI compared with ACEIs/ARBs, it would be premature to conclude ARNI is superior to ACEIs/ARBs for the management of PaHF. Given the observational design, the long study period spanning 12 years with evolving GDMT, and the potential for residual and time-dependent confounding, these findings should be interpreted with caution. More data are needed to validate our findings. Lastly, the impact of sodium-glucose cotransporter-2 inhibitors on overall mortality was not evaluated due to the limited number of patients with PaHF treated with the agent. Further studies are worth conducting regarding the efficacy of sodium-glucose cotransporter-2 inhibitors or other novel HF agents.

Nonetheless, despite these limitations, our study provides meaningful insights into the risk profile of mortality, optimal timing of CRT-upgrade, and the selection of more suitable HF medications in patients with PaHF.

## Conclusions

In this nationwide real-world cohort of patients with PaHF, CRT-upgrade and treatment with ARNI and beta-blockers were strongly associated with improved survival. Therefore, once PaHF is identified, transition to biventricular pacing in combination with contemporary HF medical therapy, preferably including ARNI and beta-blockers, should be considered.

## Supplementary Material

oeag027_Supplementary_Data

## Data Availability

Data sharing from the Authors is not possible because of legislation from the Korean government. However, additional data are available through approval and oversight by the Korean National Health Insurance Service.
