# Supplementary material for: Long-term mortality and treatment outcomes in pacemaker-associated heart failure: insights from a nationwide propensity-matched study
Source: Eur Heart J Open. 2026 Feb 16;6(2):oeag027. doi: 10.1093/ehjopen/oeag027 (PMC12962801; doi:10.1093/ehjopen/oeag027)
Supplement: oeag027_Supplementary_Data [file oeag027_supplementary_data.zip › Supple Fiugre.pptx]

## Slide 1
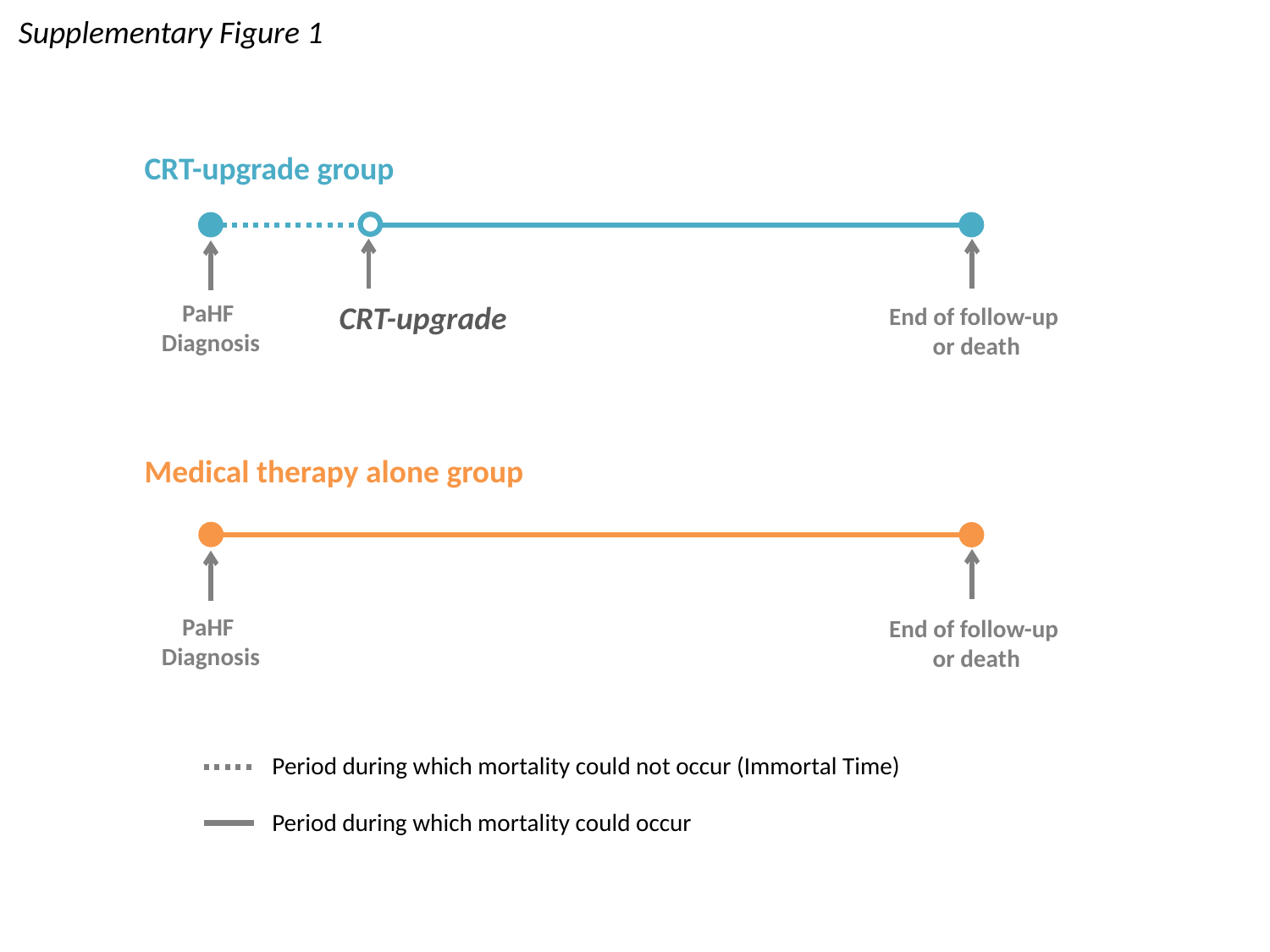

Supplementary Figure 1
CRT-upgrade group
PaHF
Diagnosis
CRT-upgrade
End of follow-up
 or death
Medical therapy alone group
PaHF
Diagnosis
End of follow-up
 or death
Period during which mortality could not occur (Immortal Time)
Period during which mortality could occur

## Slide 2
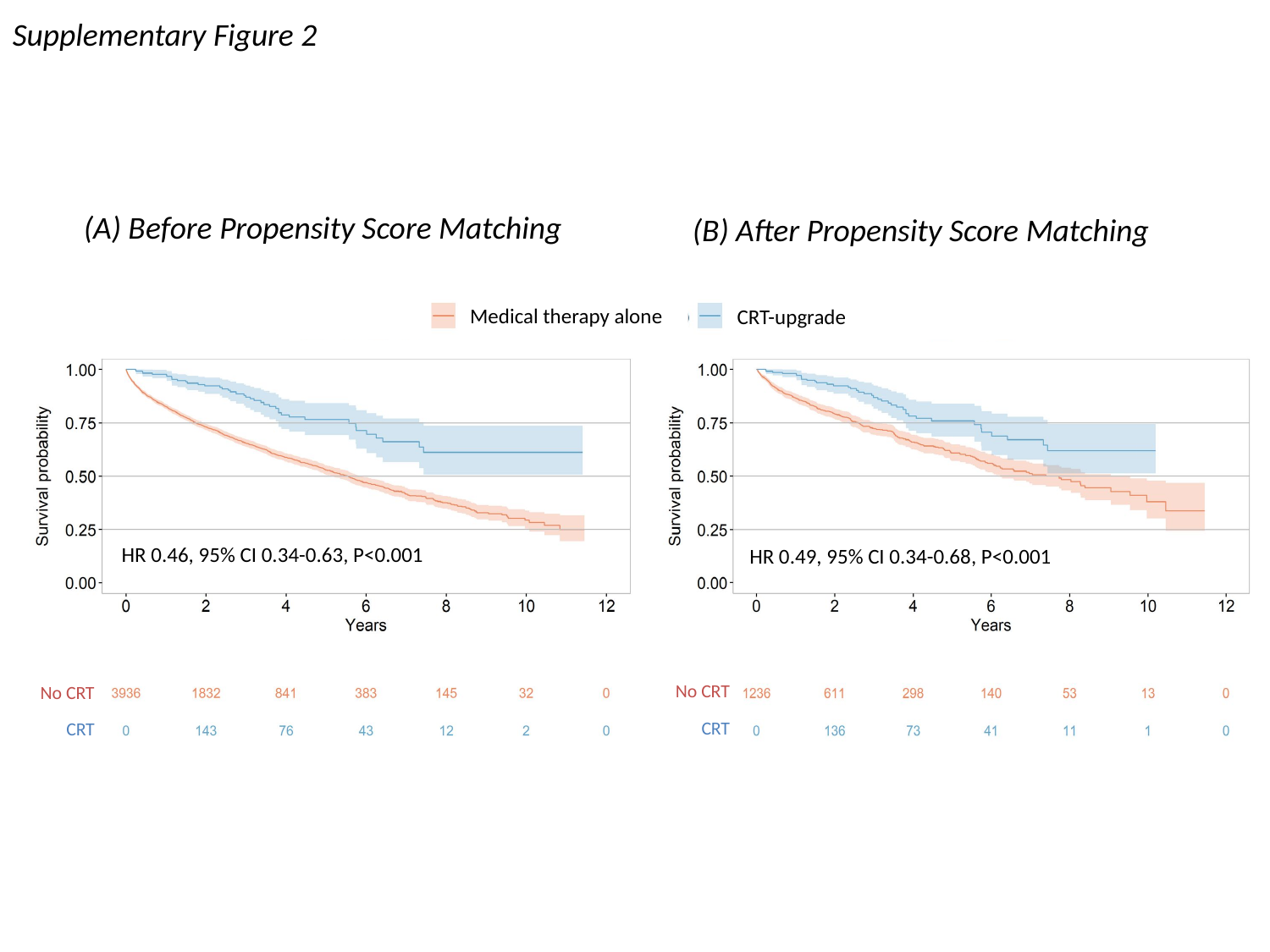

Supplementary Figure 2
(A) Before Propensity Score Matching
(B) After Propensity Score Matching
Medical therapy alone
CRT-upgrade
No CRT
No CRT
CRT
CRT
HR 0.46, 95% CI 0.34-0.63, P<0.001
HR 0.49, 95% CI 0.34-0.68, P<0.001
