# Supplementary material for: Long-term mortality and treatment outcomes in pacemaker-associated heart failure: insights from a nationwide propensity-matched study
Source: Eur Heart J Open. 2026 Feb 16;6(2):oeag027. doi: 10.1093/ehjopen/oeag027 (PMC12962801; doi:10.1093/ehjopen/oeag027)
Supplement: oeag027_Supplementary_Data [file oeag027_supplementary_data.zip › PaHF Supplement table_260119.docx]

**Supplementary Table 1.** Variable definitions by International Classification of diseases, 10^th^ revision (ICD-10) codes

| **Variable** | **ICD-10** | **Diagnostic definition** |
| --- | --- | --- |
| Diabetes | E11-E14 | Admission≥1 or outpatient department ≥ 2 |
| Hypertension | I10-I13, I15 | Admission≥1 or outpatient department ≥ 2 |
| Coronary artery disease | I20-I25 | Admission≥1 or outpatient department ≥ 2 |
| Peripheral artery disease | I70, I73 | Admission or outpatient department ≥ 2 |
| CKD/ESRD | I13.1, N03, N05, N10-N19, Z49, Z94.0, Z99.2 | Admission or outpatient department ≥ 1 |
| Valvular heart disease | I05, I06, I07, I08, I34, I35, I36, I37, I39 | Admission≥1 or outpatient department ≥ 2 |
| Atrial fibrillation | I48.0-48.4, I48.9 | Admission≥1 or outpatient department ≥ 2 |
| COPD | J41-44 | Admission or outpatient department ≥ 1 |
| AV block | I441, I442, I443 |  |
| Sinus node dysfunction | I495 |  |

Abbreviations: CKD, chronic kidney disease; ESRD, end-stage renal disease; COPD, chronic obstructive pulmonary disease; AV block, atrioventricular block.

**Supplementary Table 2.** Comorbidity definitions for Charlson comorbidity index using International Classification of Diseases, 10th revision (ICD-10) codes

| **Variable** | **ICD-10** |
| --- | --- |
| Myocardial infarction | I21.x, I22.x, I25.2 |
| Congestive heart failure | I09.9, I11.0, I13.0, I13.2, I25.5, I42.0, I42.5 - I42.9, I43.x, I50.x, P29.0 |
| Peripheral vascular disease | I70.x, I71.x, I73.1, I73.8, I73.9, I77.1, I79.0, I79.2, K55.1, K55.8, K55.9, Z95.8, Z95.9 |
| Cerebrovascular disease | G45.x, G46.x, H34.0, I60.x - I69.x |
| Dementia | F00.x - F03.x, F05.1, G30.x, G31.1 |
| Chronic pulmonary disease | I27.8, I27.9, J40.x - J47.x, J60.x - J67.x, J68.4, J70.1, J70.3 |
| Rheumatic disease | M05.x, M06.x, M31.5, M32.x - M34.x, M35.1, M35.3, M36.0 |
| Peptic ulcer disease | K25.x - K28.x |
| Mild liver disease | B18.x, K70.0 - K70.3, K70.9, K71.3 - K71.5, K71.7, K73.x, K74.x, K76.0, K76.2 - K76.4, K76.8, K76.9, Z94.4 |
| Diabetes without chronic complication | E10.0, E10.1, E10.6, E10.8, E10.9, E11.0, E11.1, E11.6, E11.8, E11.9, E12.0, E12.1, E12.6, E12.8, E12.9, E13.0, E13.1, E13.6, E13.8, E13.9, E14.0, E14.1, E14.6, E14.8, E14.9 |
| Diabetes with chronic complication | E10.2 - E10.5, E10.7, E11.2 - E11.5, E11.7, E12.2 - E12.5, E12.7, E13.2 - E13.5, E13.7, E14.2 - E14.5, E14.7 |
| Hemiplegia or paraplegia | G04.1, G11.4, G80.1, G80.2, G81.x, G82.x, G83.0 - G83.4, G83.9 |
| Renal disease | I12.0, I13.1, N03.2 - N03.7, N05.2 - N05.7, N18.x, N19.x, N25.0, Z49.0 - Z49.2, Z94.0, Z99.2 |
| Any malignancy, including lymphoma and leukemia, except malignant skin cancer | C00.x - C26.x, C30.x - C34.x, C37.x - C41.x, C43.x, C45.x - C58.x, C60.x - C76.x, C81.x - C85.x, C88.x, C90.x - C97.x |
| Moderate or severe liver disease | I85.0, I85.9, I86.4, I98.2, K70.4, K71.1, K72.1, K72.9, K76.5, K76.6, K76.7 |
| Metastatic solid tumor | C77.x - C80.x |
| AIDS/HIV | B20.x - B22.x, B24.x |

Abbreviations: AIDS, acquired immunodeficiency syndrome; HIV, human immunodeficiency virus.

**Supplementary Table 3.** Cardiac implantable electronic device and procedure definitions

| **Variable** | **Procedure codes** | **Device codes** |
| --- | --- | --- |
| **Pacemaker implantation** |  |  |
| Single chamber | O0203 | G8201, G8202 |
| Dual chamber | O0204 | G8203, G8204, G8205 |
| **ICD implantation** |  |  |
| Single chamber | O0211 | G8301 |
| Dual chamber | O0211 | G8302 |
| **CRT implantation** |  |  |
| CRT-P | O0203 or O0204 | G8103 |
| CRT-D | O0211 | G8303 |

Abbreviations: CRT-D, cardiac resynchronization therapy defibrillator; CRT-P, cardiac resynchronization therapy pacemaker; ICD, implantable cardioverter defibrillator

**Supplementary Table 4.** Risk factors of all-cause mortality, adjusting immortal-time bias in the propensity score-matched PaHF cohort

| **Variable** | ***Univariable analyses*** | | ***Multivariable model 1*** | | ***Multivariable model 2*** | |
| --- | --- | --- | --- | --- | --- | --- |
|  | **HR (95% CI)** | **P value** | **HR (95% CI)** | **P value** | **HR (95% CI)** | **P value** |
| CRT-upgrade^*^ | 0.50 (0.36 to 0.70) | <.001 | 0.49 (0.35–0.70) | <0.001 | 0.49 (0.34–0.68) | <0.001 |
| Age (per 1-year increase) | 1.05 (1.04 to 1.06) | <.001 | 1.05 (1.04–1.06) | <0.001 | 1.05 (1.04–1.06) | <0.001 |
| Male | 1.19 (0.98 to 1.45) | 0.079 | 1.36 (1.12–1.65) | 0.002 | 1.39 (1.14–1.68) | <0.001 |
| Diabetes mellitus | 1.45 (1.18 to 1.78) | <.001 | 1.28 (1.02–1.60) | 0.034 | 1.28 (1.02–1.60) | 0.034 |
| Hypertension | 0.60 (0.48 to 0.76) | <.001 | 0.50 (0.37–0.67) | <0.001 | 0.45 (0.33–0.61) | <0.001 |
| Coronary artery disease | 1.21 (0.99 to 1.48) | 0.064 | 1.08 (0.87–1.33) | 0.498 | 1.06 (0.86–1.31) | 0.603 |
| Peripheral artery disease | 1.20 (0.97 to 1.47) | 0.088 | 1.01 (0.81–1.25) | 0.932 | 1.01 (0.81–1.25) | 0.961 |
| CKD/ESRD | 2.22 (1.76 to 2.81) | <.001 | 1.71 (1.34–2.19) | <0.001 | 1.68 (1.31–2.15) | <0.001 |
| Valvular heart disease | 0.76 (0.57 to 1.01) | 0.058 | 1.04 (0.79–1.38) | 0.766 | 1.03 (0.78–1.37) | 0.830 |
| Atrial fibrillation | 0.72 (0.58 to 0.90) | 0.004 | 0.89 (0.69–1.15) | 0.367 | 0.91 (0.71–1.18) | 0.483 |
| COPD | 1.38 (1.13 to 1.69) | 0.002 | 1.15 (0.94–1.40) | 0.169 | 1.14 (0.94–1.39) | 0.183 |
| RAS inhibitors**^†^** | 0.59 (0.48 to 0.73) | <.001 | 0.71 (0.57–0.89) | 0.003 | — | — |
| ACEIs or ARBs | 0.71 (0.58 to 0.87) | <.001 | — | — | 0.84 (0.66–1.06) | 0.141 |
| ARNI | 0.30 (0.16 to 0.56) | <.001 | — | — | 0.39 (0.20–0.76) | 0.005 |
| MRAs | 1.14 (0.92 to 1.40) | 0.224 |  |  |  |  |
| Beta blockers | 0.66 (0.54 to 0.81) | <.001 | 0.77 (0.62–0.97) | 0.024 | 0.78 (0.63–0.98) | 0.029 |

*, CRT-upgrade was incorporated into the models as a time-dependent covariate of ‘time from PaHF diagnosis to CRT-upgrade’.

**†**, The variable for RAS inhibitors (ACEIs/ARBs and ARNI) was included as a single composite variable in Model 1, whereas ACEIs/ARBs and ARNI were included separately in Model 2.

Abbreviations as shown in Tables 1 and 2.
